# The effect of short-term preoperative nutritional intervention for cleft surgery eligibility

**DOI:** 10.1186/s40795-023-00704-1

**Published:** 2023-03-14

**Authors:** Shady Mikhail, Lily Chattopadhyay, Melissa DiBona, Charlotte Steppling, Dede Kwadjo, Anjaramamy Ramamonjisoa, Wendy Gallardo, Fatima Almendarez, Beau Sylvester, Samanta Rosales, Ibrahim Nthalika, Zachary J. Collier, William Magee, Allyn Auslander

**Affiliations:** 1 Operation Smile, Inc, 3641 Faculty Blvd, Virginia Beach, VA 23453 USA; 2 Children’s Hospital Los Angeles, Los Angeles, CA USA; 3 Operation Smile Ghana, Accra, Ghana; 4 Operation Smile Madagascar, Antananarivo, Madagascar; 5 Operation Smile Venezuela, Caracas, Venezuela; 6 Operation Smile Nicaragua, Managua, Nicaragua; 7 Operation Smile Honduras, Tegucigalpa, Honduras; 8 Operation Smile Malawi, Lilongwe, Malawi; 9 California Division of Plastic & Reconstructive Surgery, University of Southern, Los Angeles, CA USA

**Keywords:** Orofacial clefts, Cleft lip and palate, Pediatrics, Malnutrition, Low- and middle- income countries, Ready-to-use therapeutic foods

## Abstract

**Background:**

Children with orofacial clefts are highly susceptible to malnutrition, with severe malnutrition restricting their eligibility to receive safe surgery. Ready-to-use therapeutic foods (RUTF) are an effective treatment for malnutrition; however, the effectiveness has not been demonstrated in this patient population prior to surgery. We studied the effectiveness of short-term RUTF use in transitioning children with malnutrition, who were initially ineligible for surgery, into surgical candidates.

**Methods:**

A cohort of patients from Ghana, Honduras, Malawi, Madagascar, Nicaragua, and Venezuela enrolled in a nutrition program were followed by Operation Smile from June 2017 to January 2020. Age, weight, and length/height were tracked at each visit. Patients were included until they were sufficiently nourished (Z >  = -1) with a secondary outcome of receiving surgery. The study was part of a collaborative program between Operation Smile (NGO), Birdsong Peanuts (peanut shellers and distributors), and MANA Nutrition (RUTF producer).

**Results:**

A total of 556 patients were recruited between June 2017 and January 2020. At baseline 28.2% (*n* = 157) of patients were diagnosed with severe, 21.0% (*n* = 117) moderate, and 50.7% (*n* = 282) mild malnutrition. 324 (58.3%) presented for at least one return visit. Of those, 207 (63.7%) reached optimal nutrition status. By visit two, the mean z-score increased from -2.5 (moderate) to -1.7 (mild) (*p* < 0·001). The mean time to attain optimal nutrition was 6 weeks. There was a significant difference in the proportion of patients who improved by country(*p* < 0.001).

**Conclusion:**

Malnutrition prevents many children with orofacial clefts in low- and middle-income countries from receiving surgical care even when provided for free. This creates an even larger disparity in access to surgery. In an average of 6 weeks with an approximate cost of $25 USD per patient, RUTF transitioned over 60% of patients into nutritionally eligible surgical candidates, making it an effective, short-term preoperative nutritional intervention. Through unique partnerships, the expansion of cost-effective, large-scale nutrition programs can play a pivotal role in ensuring those at the highest risk of living with unrepaired orofacial clefts receive timely and safe surgical care.

**Supplementary Information:**

The online version contains supplementary material available at 10.1186/s40795-023-00704-1.

## Background

The need for safe, essential surgical care in low- and middle- income countries (LMICs) has been clearly established with an estimated 5 billion people lacking access to timely, affordable, and effective surgery of any kind [1]. The surgical burden of congenital conditions, such as orofacial clefts (OFC), is often exacerbated not only by the lack of access to surgery, but surgical ineligibility due to existing comorbidities such as malnutrition even when care is available [2, 3]. OFCs are among the most common congenital birth defects globally with an incidence of 1 in 700 live births [4, 5]. Pediatric patients with OFCs are more likely to suffer from malnutrition than healthy children [6] with a prevalence of malnutrition cited as high as 30–50% globally [7, 8].

Malnutrition in children with OFCs is primarily due to feeding difficulties, including the inability to generate enough suction pressure during breastfeeding or artificial nipple feeding, as well as the inability to organize and retain the food bolus within the oro-pharyngeal cavities before swallowing [9]. Methods for assisted feeding, such as squeezable bottles [10] and modified nipples [9], are often recommended for OFC patients; however, these options are often unavailable in LMICs. Additionally, due to the lack of access to comprehensive medical services and clean water sources in LMICs, caretakers often are not provided with the proper knowledge, training, and/or feeding supplies necessary to fulfill the nutritional needs of children with OFCs [11, 12].

Malnutrition leads to an increased likelihood of wound healing complications and infection due to immunodeficiency which leads to an increased risk of postsurgical complications and need for further operation [13, 14]. Several feeding interventions for pediatric OFC patients have been described in the literature, but none discuss the impact of these approaches on surgical eligibility and/or outcomes. A 1994 study in the UK established the positive impact of implementing an early feeding program for infants with OFC to reduce failure to thrive and improve weight gain [9, 15]. However, the study did not assess the benefits of their intervention in reaching surgical eligibility and, due to resource constraints, is not generalizable to LMICs.

Although preoperative nutritional intervention programs have not been explored in patients with OFC, the benefits of these programs in improving surgical outcomes have been explored in other patient populations. In a study of patients with head and neck cancer and malnutrition, the patients were given an immuno-nutrition (IN) solution which resulted in a significantly lower rate of postoperative complications and length of hospital stay [16]. In a study on 512 patients who had undergone abdominal surgery, they demonstrated the benefit of enteral and parenteral nutritional compounds in lowering postoperative complication rates and length of hospital stay in comparison to the control group (25.6% vs. 50.6% and 13.7 vs. 17.9 days, respectively) [17].

A current approach to combat malnutrition is through ready-to-use therapeutic foods (RUTF), which have been demonstrated to be an effective treatment for children with moderate and severe malnutrition, especially in LMICs and emergency settings [18]. RUTF is a peanut paste that is high in proteins, carbohydrates, lipids, and other nutrients providing a home-based treatment that has a long shelf life, does not require refrigeration or cooking, and has a very low water content making it resistant to bacterial growth [18]. Six unique studies performed in Malawi [19–22], Niger [23], and Sierra Leone [24] have shown that RUTF supplementation exhibited significantly higher rates of recovery from malnutrition and greater average weight gain compared to the control patients receiving other non-RUTF supplements, such as corn-soy blend or the high-energy milk-based liquid foods, F-75 and F-100.

The benefits of preoperative nutritional interventions for patients to reach surgical eligibility have been minimally studied in the non-cleft pediatric population and they have not been at all studied with respect to patients with OFC. This study assesses the benefits of preoperative nutritional supplements with respect to the pediatric OFC population in LMICs. Specifically, this study will assess the outcomes of providing RUTF to treat patients with mild, moderate, and severe malnutrition who were previously deemed ineligible for cleft lip and/or cleft palate surgery to reach surgical candidacy with respect to time required and Z- score changes. The findings of this program, which spans from LMICs in three continents, have the potential to foster improved pediatric nutritional approaches in LMICs and the development of affordable, preoperative nutritional intervention programs to ensure both surgical eligibility and improved surgical outcomes of patients with OFC.

## Methods

### Aim

Assess the benefits of the short-term preoperative administration of RUTF nutritional supplement on improving surgical eligibility for patients with OFC being treated by Operation Smile (OS).

### Study design and setting

This study followed a cohort of malnourished pediatric patients with OFC in Ghana, Honduras, Madagascar, Malawi, Nicaragua, and Venezuela. Data for this assessment was collected from 2017 through January 2020 as part of a collaborative nutrition program between: OS, an internationally recognized not-for-profit organization that has been providing free cleft surgical care to patients for over 37 years; Birdsong Peanuts, a leading peanut shelling and distribution company in the U.S.; and MANA Nutrition, a U.S.-based producer of RUTF. Patient data for the study was collected during 34 surgical missions (2 in Nicaragua, 9 in Madagascar, 13 in Ghana, 4 in Honduras, and 6 in Malawi) and continuously at 6 dedicated cleft care centers (1 in Nicaragua, 1 in Honduras, 2 in Venezuela, and 2 in Malawi). The majority of the data in Malawi was collected at the end of 2019/ early 2020 resulting in minimal follow-up data available. Detailed information related to mission locations and sites are listed in Additional file 1. A retrospective review of the existing OS programmatic data was IRB approved by Children’s Hospital Los Angeles (CHLA). All necessary country level approvals were obtained by Operation Smile prior to initiating the program by in-country staff members.

### Study population

The study included all patients with OFC and malnutrition, aged 6 months to 19 years, who presented at OS surgical mission sites and/or care centers indicated in Additional file 1. OFCs include cleft lip only (CLO), cleft lip and palate (CLP), or cleft palate only (CPO) with the defect ranging from a small notch in the lip to a complete opening through the lip, floor of the nose, and palate. ICD10 codes 35–37 were used for this diagnosis and confirmed by a medical practitioner [25]. Patients were enrolled in the nutritional program if they were not eligible (Z <  = -2) or not optimal candidates (Z <  = -1) for surgery due to malnutrition as defined by Weight-for-Length Z-score (WLZ) or Weight-for-Height Z-Score (WHZ < -). The severity of malnutrition was stratified into three tiers: mild (z-score: -1 to > -2), moderate (z-score: -2 to > -3), and severe (z-score: ≤ -3). Patients were excluded from the nutrition program if they were immediately eligible for surgery, did not qualify as an OS surgical candidate for reasons besides nutrition, or were allergic to peanuts or any other ingredients in the RUTF. All patients who were enrolled in the program and met inclusion criteria were included in the analysis. No allergies were identified or exhibited in any eligible participants. Nutritional characteristics of the patients were identified using the World Health Organizations (WHO) weight-for-length or weight-for-height standards [26].

### Description of RUTF

The RUTF supplement is a low moisture content, peanut butter-based paste that does not require refrigeration or mixing with any other substances prior to consumption, has a shelf life of approximately 2 years, and is safe for patients as young as 4 months [27]. Each 106-g sachet contains approximately 500 kilo calories, 12.5 g of protein, 29 g of fat, and 47 g of carbohydrates in addition to micronutrients such as zinc, folic acid, and other vitamins and minerals [28]. A wide variety of efficacy studies looking at RUTF to treat malnutrition have been previously published [20, 21, 27, 29].

### Data collection and management

At each mission site and center, a pediatrician evaluated the patients to determine the nutritional status after the patient’s cleft diagnosis had been established. Following the medical screening process, if patients were ineligible for surgery due to malnutrition, they were enrolled in the nutrition assistance program by in-country medical team members. Information regarding the nutrition program and RUTF supplements was explained to the primary caretakers in their native language by bilingual healthcare staff. Informed consent was obtained from the patient’s caretakers before the child was enrolled in the program. No caretakers or patients declined participation. The families were then taken to a private or semi-private area to complete a baseline questionnaire. The questionnaire included the patient’s baseline anthropometric measurements of weight and height or length.

Upon completion of the questionnaire, the patients were given the RUTF sachets. The number of RUTF sachets distributed differed by patient and was determined by the pediatrician depending on the weight/height recommended dose, severity of the malnutrition, how far the patient lived from the center, and how often the patient was able to return for a follow-up visit. Families were advised to return for follow-up visits every two weeks, however, as most patients lived far from the centers, that was not always possible. If they were unable to return for their scheduled visits, the staff attempted to contact the family by phone multiple times to check on the patient’s RUTF supply and nutritional changes. When feasible, the local staff delivered more RUTF sachets to the patient if they required more but were unable to visit the center. Patients stopped receiving RUTF when they were deemed no longer malnourished and were eligible for surgery, or when coordinators were unable to contact or follow-up with the patients.

### Statistical analysis

WHO defines moderate and severe pediatric malnutrition as a WHZ or WLZ of -2 to > -3 and ≤ -3, respectively. For the purposes of stratification in this study, mild malnutrition was defined as a WHZ or WLZ of -1 to > -2 per the AND/ASPEN consensus. The WLZ was used for children 0 to 23 months and WHZ for children 24 to 59 months of age. The Academy of Nutrition and Dietetics (AND) also uses the same z-score parameters as WHO for BMI-for-age for children 2 to 20 years old to define the severity of malnutrition [30, 31].

WHZ, WAZ, HAZ, and BMI-for-age z-scores were determined using their respective WHO z-score paper charts for girls and boys. OS uses the WHO standards in determining the nutritional status of patients with OFC and their eligibility for surgery. Descriptive statistics, including means and proportions were used for the demographic characteristics for the patients with OFCs and their caretakers. Tests of statistical significance included t-tests for continuous variables and chi-squared tests for categorical data. Results were considered statistically significant if *p*-values were less than 0.05. Microsoft Excel was used for the data analysis.

## Results

### Population characteristics and magnitude of malnutrition

**Fig. 1:**
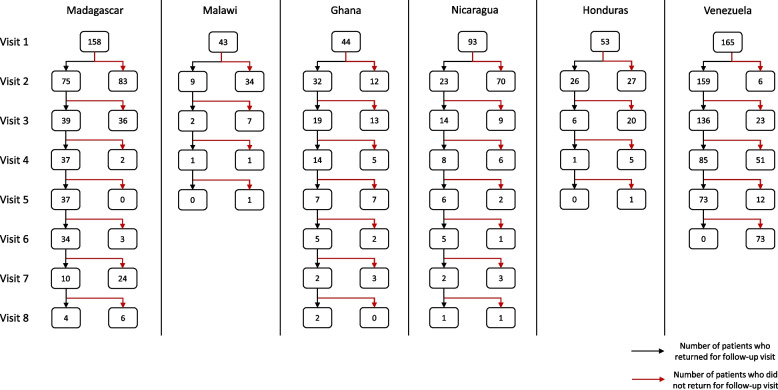
The number of patients per visit per country

Table 1 provides a demographic breakdown of the patients in the six study countries (Ghana, Honduras, Madagascar, Malawi, Nicaragua, and Venezuela). Between 2017 and 2020, a total of 677 patients were admitted into the RUTF program. In total, 121 patients were excluded from the evaluation of the program due to insufficient data, leaving a total of 556 patients for analysis with an average baseline age of 37 months (range 6, 225 months). Overall, 35.3% of patients were a year or younger, 27.2% were between 1- 2 years, 21.0% between 2- 5 years, and 16.5% over five-years old; however, this distribution significantly differed by country. There were 324 (58.3%) patients who presented for at least one return visit. Figure 1 shows the number of patients at baseline and their subsequent visits by country. The male-to-female ratio was approximately 1:1. The most prevalent type of OFC was cleft lip and palate (CLP) (47.4%), followed by cleft lip only (CLO) (37.7%), and cleft palate only (CPO) (13.5%). At baseline, a total of 157 (28.2%) patients were diagnosed with severe malnutrition, 117 (21.0%) with moderate malnutrition, and 282 (50.7%) with mild malnutrition. This differed significantly by country (*p* < 0.001 for all groups) with Venezuela having the largest percentage of patients with severe malnutrition (37%) and Nicaragua having the smallest (10.8%). Patients received an average of forty RUTF sachets per six- week period (range: 8–141 total sachets).

**Table 1:** Demographic characteristics of RUTF patients (*N* = 556)

	**Madagascar (*n* = 158)**	**Malawi (*n* = 43)**	**Ghana (*n* = 44)**	**Nicaragua (*n* = 93)**	**Honduras (*n* = 53)**	**Venezuela (*n* = 165)**	**Total (*n* = 556)**
Sex (%M)	50.6%	48.8%	34.1%	55.9%	43.4%	55.2%	51.6%
Avg age (months) (std.)	17.6 (13.1)	11.1 (5.0)	13.7 (6.5)	61.22 (55.1)	37.2 (37.6)	51.2 (42.9)	35.9 (39.9)
Age Distribution (months)							
< 12 mos	75 (47.5%)	29 (67.4%)	25 (56.8%)	12 (12.9%)	10 (18.9%)	45 (27.3%)	196 (35.3%)
12- 24 mos	57 (36.1%)	13 (30.2%)	17 (38.6%)	19 (20.4%)	20 (37.7%)	25 (15.2%)	151 (27.2%)
24- 60 mos	24 (15.2%)	1 (2.3%)	2 (4.5%)	36 (38.7%)	14 (26.4%)	40 (24.2%)	117 (21.0%)
60 + mos	2 (1.3%)	0 (0%)	0 (0%)	26 (28.0%)	9 (17.0%)	55 (33.3%)	92 (16.5%)
Malnutrition Status:							
*Mild*	71 (44.9%)	16 (37.2%)	11 (25.0%)	73 (78.4%)	26 (49.1%)	85 (51.5%)	282 (50.7%)
*Moderate*	40 (25.4%)	13 (30.2%)	20 (45.5%)	10 (10.8%)	15 (28.3%)	19 (11.5%)	117 (21.1%)
*Severe*	47 (29.7%)	14 (32.6%)	13 (29.5%)	10 (10.8%)	12 (22.6%)	61 (37.0%)	157 (28.2)
Avg. # of RUTF Sachets (N (Range))	48 (15–141)	45 (11–84)	50 (30–78)	43 (12–117)	42 (15–100)	28 (8–63)	40 (8–141)
# Surgeries Confirmed	44	24	28	26	12	61	195

### Outcomes of RUTF administration

Of the 324 patients who returned for at least one visit, 207 (63.7%) patients who were previously malnourished were no longer malnourished (Z >  = -1). Of those 207 patients, 32 (15.5%) were initially diagnosed with severe malnutrition, 30 (14.5%) with moderate malnutrition, and 145 (70.0%) with mild malnutrition. The average time to reach surgical eligibility in this group was 6 weeks with a range of 1–103 weeks (Table 2).

**Table 2:** Direct outcomes of administering RUTF on all patients who returned for at least one follow-up visit (*N* = 324)

	**Madagascar (*n* = 158)**	**Malawi (*n* = 43)**	**Ghana (*n* = 44)**	**Nicaragua (*n* = 93)**	**Honduras (*n* = 53)**	**Venezuela (*n* = 165)**	**Total (*n* = 556)**
# adequately nourished (Z > = -1)	72 (45.6%)	2 (4.6%)	11 (25%)	16 (17.2%)	6 (11.3%)	100 (60.6%)	207 (37.3%)
**Initial malnutrition status of patients recovered:**							
Mild	43 (59.7%)	1 (50.0%)	7 (63.6%)	13 (81.3%)	6 (100.0%)	75 (75.0%)	145 (70.0%)
Moderate	15 (20.8%)	1 (50.0%)	4 (36.4%)	1 (6.3%)	0 (0.0%)	9 (9.0%)	30 (14.5%)
Severe	14 (19.5%)	0 (0.0%)	0 (0.0%)	2 (12.5%)	0 (0.0%)	16 (16.0%)	32 (15.5%)
Avg time to eligibility (weeks) (range)	2.4 (1–30)	27.8 (26–30)	11.0 (2–40)	8.6 (2–43)	27.3 (3–103)	6.8 (1.5–30)	6.4 (1–103)

**Fig. 2:**
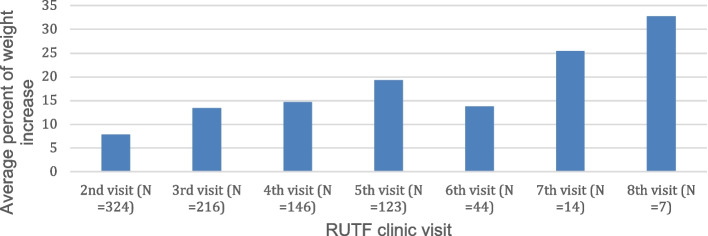
Average percent increase in weight over course of RUTF treatment

Figure 2 shows the percentage of weight change for patients who returned for at least one visit (*n* = 324). Overall, at their first follow-up visit patients gained an average of 7.8% of their initial body weight. The average percentage of weight gain increased per visit as patients received more RUTF and additional follow-up visits. Patients tracked through seven follow-up visits (*n* = 7, range 7–43 weeks) increased an average of approximately 33% of their initial body weight. A strong, positive correlation was observed between the number of follow-up visits and the average percent increase in weight (*r* = 0.89).

**Fig. 3:**
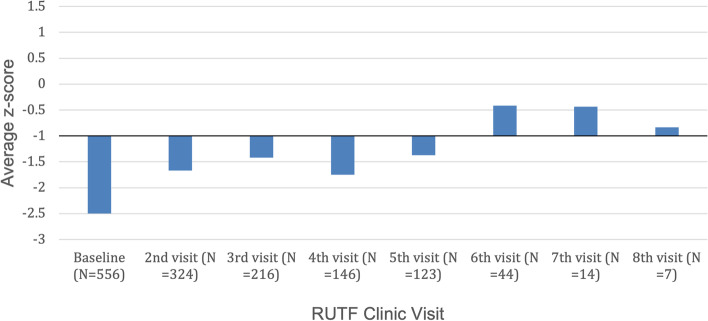
Average z-score over the course of the RUTF treatment

Figure 3 summarizes the change in the mean z-score (either WHZ or WLZ depending on patient age), and thus the average nutritional status, by visit. The mean z-score of all the patients at baseline was within the moderately malnourished range (-2 to > -3). By the second visit, the mean z-score increased from -2.5 to -1.7 (*p* < 0.001). There was also an overall positive correlation between the number of visits and the increase in the average z-score (*r* = 0.87). By the 6th visit, the mean z-score for the 44 returning patients was in the normal range (z > -1), indicating they were no longer malnourished.

**Fig. 4:**
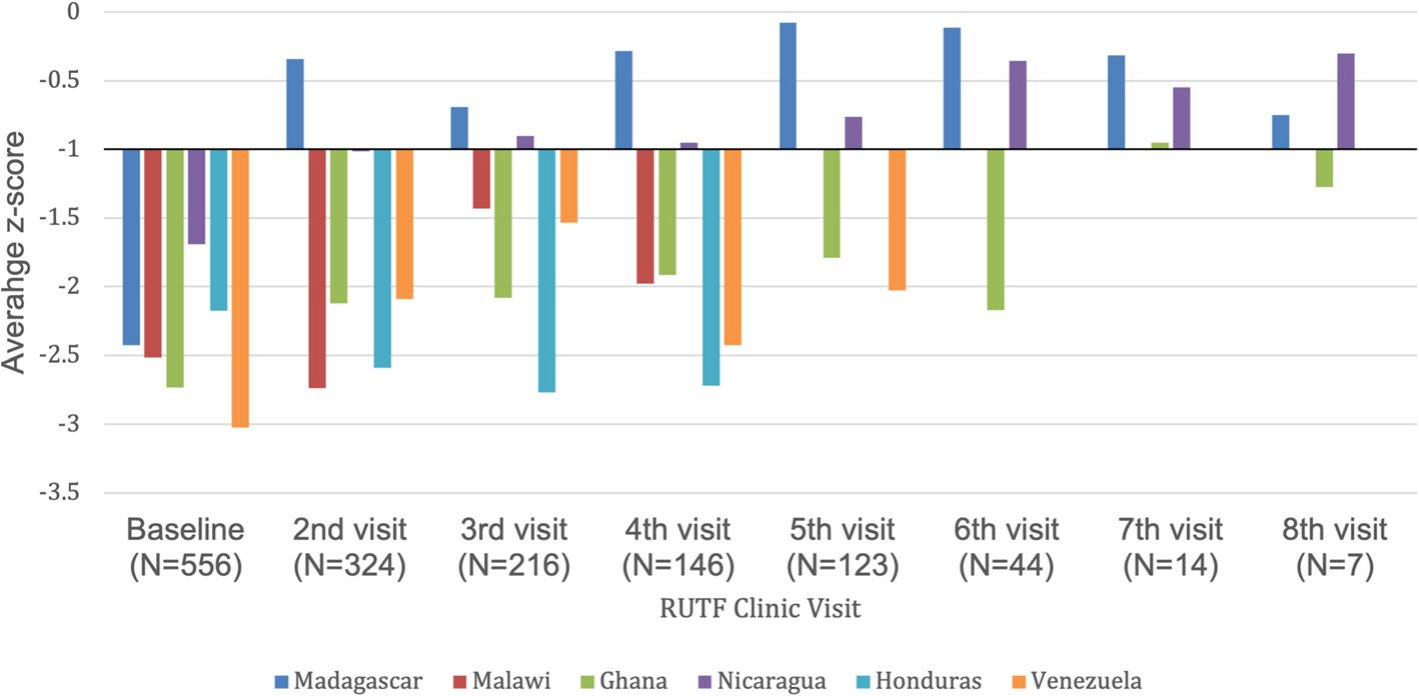
Average z-score by country over the course of the RUTF treatment

The change in the average z-score by country is shown in Fig. 4. In Madagascar (*n* = 158), there was a significant overall improvement in the combined mean z-score from -2.4 to -0.34 demonstrated as early as the 2nd visit (*p* < 0.001), and it remained within the normal range for all subsequent visits. In Nicaragua (*n* = 93), there was also a statistically significant improvement in z-score by the 2nd visit as the average z-score increased from -1.7 to -1.0 (*p* = 0.003). In Ghana, the mean z-score increased significantly from -2.7 to -1.9 by the 2nd visit (*p* = 0.023), and to normal (> -1) for those who reached the 7th follow-up visit. While not statistically significant, there was an increase in the average z-score in Nicaragua from -1.0 on the 2nd visit to -0.9 (normal) on the 3rd visit. In Malawi, Honduras, and Venezuela, the maximum number of follow-up visits were not sufficient to detect an effect (*n* = 4, 4, and 5, respectively).

## Discussion

Given the extensive number of barriers that exist for children from LMICs needing surgical treatment of OFCs, it is critical that we understand how to make surgery as safe and effective as possible when it finally becomes available to them. Operation Smile (OS), a globally active non-governmental organization providing cleft surgery and comprehensive care, has supplied and tracked the distribution of RUTF supplementation to 677 patients in six LMICs along with the patients’ weight gain to determine their nutrition status and eligibility to receive cleft surgery. The program demonstrated the significant benefits of a short-term preoperative nutritional intervention on nutritional status and surgical eligibility in a large trans-continental group of pediatric patients with OFC in LMICs. While studies have previously established the significant impact of RUTF on improving the nutritional status of children with malnutrition [19, 27, 32], the focus of our study on a large population of potential surgical patients with OFC with malnutrition is unique in the literature. This knowledge is pertinent because patients with OFC are an especially vulnerable population, as systemic conditions in LMICs may exacerbate malnutrition even in patients without OFC, putting patients with disease at a greater disadvantage [33]. This impactful program uniquely addresses pediatric malnutrition from a collaborative standpoint through a cost-effective, short-term, and context-appropriate solution for individuals in LMICs.

Based on the global standards of care used by OS, nearly two-thirds of the initially malnourished patients who returned for follow-up visits were eligible for cleft surgery at the end of their RUTF treatment. This corroborates the existing evidence that RUTF packets are very effective in resource-constrained settings where surgical NGOs frequently operate [18, 19, 24]. As patients are often sequenced into care prior to the surgical mission by OS in-country teams, this program will provide a highly effective treatment to ensure that when children present for surgery, they are not unnecessarily delayed by treatable causes since these delays in surgical care often lead to lifelong disability. Short-term programs such as these can be used with minimal contact to patients prior to surgery (for example, 1 visit 6 weeks prior) to ensure that they are adequately nourished when the surgical teams are in-country and available to provide treatment.

It has been demonstrated that patients with OFC are more prone to malnutrition (30%- 50% of all cases) than patients without OFC [7, 8], which translates to a minimum of 30% of all patients with OFC globally requiring nutritional intervention. The baseline prevalence of mild, moderate, and severe malnutrition among patients with OFC in our program were 51%, 21%, and 28%, respectively. Of the 207 patients who returned for follow-up and were eligible for surgery, the majority (70%) had mild malnutrition at baseline, and the remainder were equally split between moderate and severe malnutrition. Despite the high prevalence of malnutrition in OFC patients, the majority of the patients enrolled in this program were mild cases. As a result, they were quickly brought to a healthy weight to receive surgery without intensive or complex nutritional intervention and monitoring. This knowledge can help streamline processes in the future and conserve in-country nutrition resources for patients with more severe needs.

Funding is a critical component of determining sustainability for programs in LMICs. The RUTF program was both designed and proven to be a cost-effective intervention. According to the market value of RUTF, the average cost per patient of RUTF treatment using product-dosing guidelines of two to three sachets per day for a two-month period is approximately $45. When considering the severity of the diagnosis and needs of the patients with OFC to get to the threshold of surgical eligibility, using -2-one-to-two-week treatment periods could be effective for a large portion of patients. This means the cost of RUTF nutritional intervention could be reduced to as low as $10 per patient depending on severity, prior to negotiated supply chain arrangements.

The partnership model used in this program reduced costs even further, which can be utilized as a strategy for similar interventions by non-profits and LMIC groups. The success of the collaboration between local communities, the private sector (Birdsong Peanuts and MANA), and a non-profit organization (OS) counterbalanced costs for added programmatic infrastructure such as medical oversight and patient outreach. Outside of economic benefits, these partnerships have made it possible to establish an extensive nutrition program spanning six countries and three continents with consistent, sustainable standards of care and data collection methods. This improved efficiency has facilitated research, which is often lacking yet critical to the improvement of programmatic interventions in LMICs.

We noted many country- level differences that should be explored further as this program continues to develop. The age structure of patients enrolled differed by country. Individuals enrolled in Malawi, Madagascar and Ghana were younger with the majority being less than two-years-old. Those in Honduras, Nicaragua, and Venezuela tended to be older with 17- 33% of individuals being greater than five-years-old (compared to 1% or less in the African countries). This difference is most likely attributed to programmatic differences. The Latin American countries have center-based care that runs continuously all year long. This means that many patients are found early (prior to being old enough for surgery and RUTF) and enrolled in other nutrition programs to ensure they maintain a healthy weight. In the African countries, many patients are screened on the surgical programs once they are eligible age-wise (> 6 months) thus they expect to enroll higher numbers of young patients who are malnourished being seen for the first time. As this program expands, we hope to be able to further elaborate on these differences and how it should affect the strategy in each country. However, as RUTF has been shown to be effective to combat malnutrition in all age categories, it appears to be a solution regardless of age.

The main limitation of the program, as is true for most research in LMICs, is the difficulty of ensuring long-term follow-up care. In all six countries, a large proportion of patients live far from the OS cleft centers, creating geographical and financial barriers. In many cases, even with travel costs supplemented by OS, patients are faced with the challenges of inadequate or unsafe roads, inconsistent transportation, and extensive travel times making it exceedingly difficult to seek biweekly follow-up care. To further complicate long-term care, communication with families is challenging in these settings. Many families lack access to personal phones, cell service, or electrical power, making it difficult to reach patients to reschedule appointments or to conduct follow-ups by phone. However, the large number of patients who became eligible for surgery after one follow-up visit supports the idea that a single baseline visit to receive the needed supplement could suffice in situations where follow-up is not possible. Additionally, as the program was not designed for comparative research between countries, country level differences require further elucidation. Operation Smile runs autonomously within each country and therefore has different recruitment strategies, geographic reach, and relationships with existing health infrastructure as well as other NGOs. Further research will help better understand these differences and their programmatic impact.

A common concern when considering supplementary feeding programs is the possibility that other family members consume the food intended for the patient, also known as leakage [19]. In our study, leakage was not tracked, but families were strongly advised to adhere to the prescribed feeding plan for their children to receive surgery, which we believe highly reduced the likelihood of leakage. However, if leakage was an issue, it would cause an underestimation of weight gain per RUTF consumption meaning that the program would be even more effective than calculated. Other significant and recent limitations to providing timely surgical care are the COVID-19 pandemic and related surgical restrictions, which accounts for approximately one third of our study period. The number of surgeries performed would likely have been higher had surgical programs not been halted by the pandemic.

The pilot and initial data collection has led to new developments that will inform the future directions of the program. To improve our ability to consistently provide RUTF supplement to patients and track their progress, we will explore expanding partnerships with local, community healthcare organizations in the field. Strengthening the local healthcare systems and training community health workers to manage the distribution of RUTF and follow-ups will also expand our network and allow us to have a better understanding of what is required for local patients with OFC to achieve surgical eligibility. Additionally, because our study did not include infants younger than 6 months old and did not assess assisted-feeding interventions, a future study using a cohort under 6 months of age will be conducted to assess effective feeding methods in reducing malnutrition during infancy. Further work is also needed to monitor adherence to supplementary feeding plans and track the patients postoperatively to assess any effect that short-term nutrition interventions may have on surgical outcomes.

## Conclusions

Innovative programs that successfully treat malnutrition in OFC patients are imperative as they ensure that this vulnerable patient population receives timely surgical care which improves their overall health and quality of life. The outcome of Operation Smile’s global RUTF program demonstrates that a short-term preoperative nutritional intervention can be highly effective in reducing malnutrition rates in children with OFCs so they may receive life-changing surgery.

The findings presented have the potential to reform the current approaches to malnutrition in pediatric patients with OFC in LMICs. The high number of patients diagnosed with mild malnutrition highlights that there is a large group of patients with OFC that solely require a short-term, non-resource intensive intervention to become eligible for surgery– leaving little reason for them to miss any opportunity to receive care. Furthermore, organizations that already have extensive in-country infrastructure and patient tracking systems in place are well-equipped to provide targeted, large-scale nutrition intervention programs based on this model.

It will be critical to establish longer term follow-up for patients with moderate and severe malnutrition to better understand the time required to adequately treat those more severe cases; however, these programs can now be utilized for those most in need as most of the population with OFC are expected to benefit.

Considering the medical efficacy of RUTF, its low cost, long shelf life, and well-tolerated taste, programs utilizing RUTF show great promise for treating OFC patients suffering from malnutrition so they may receive surgical care to mitigate the lifelong physical, emotional, and mental impact of having an unrepaired cleft.

## Supplementary Information

**Additional file 1.**

## Data Availability

The datasets generated and/or analyzed during the current study are not publicly available due to Operation Smile policies but are available from the corresponding author on reasonable request.

## Abbreviations

OFCs: Orofacial clefts
RUTF: Ready-to-use therapeutic foods
CLO: Cleft lip only
CLP: Cleft lip and palate
CPO: Cleft palate only
WHO: World Health Organizations
WLZ: Weight-for-Length Z-score
WHZ: Weight-for-Height Z-Score
OS: Operation Smile
