# Supplementary material for: The effect of short-term preoperative nutritional intervention for cleft surgery eligibility
Source: BMC Nutr. 2023 Mar 14;9:47. doi: 10.1186/s40795-023-00704-1 (PMC10012294; doi:10.1186/s40795-023-00704-1)
Supplement: Supplementary file 1 — Additional file 1. [file 40795_2023_704_MOESM1_ESM.docx]

**Additional file 1.** All Sites Used for Patient Recruitment and Follow-up Visits

| **Country** | **City** | **Site** |
| --- | --- | --- |
| Honduras | Tegucigalpa | Hospital San Felipe and Hospital María de Especialidades Pediátricas |
|  | San Pedro Sula | Hospital Leonardo Martínez |
|  | Santa Rosa de Copan | Hospital de Occidente |
|  | Comayagua | Hospital Regional Santa Teresa |
|  | Tegucigalpa | Clínica de Operación Sonrisa, Honduras |
| Ghana | Ho | Ho Teaching Hospital |
|  | Tamale | Tamale Teaching Hospital |
|  | Koforidua | Eastern Regional Hopsital |
|  | Dodowa | Shai Osu Doku District Hopsital |
|  | Cape Coast | Capecoast Teaching Hospital |
|  | Accra | Korle Bu Teaching Hospital |
|  | Accra | Greater Accra Regional Hospital |
| Madagascar | Tamatave | Centre Hospitalier Universitaire Analankininina |
|  | Antsirabe | Centre Hospitalier de Référence Régionale du Vakinankaratra |
|  | Tana | Centre Hospitalier Universitaire Joseph Ravoahangy Andrianavalona |
|  | Majunga | Centre Hospitalier Universitaire Professeur Zafisaona Gabriel |
| Malawi | Blantyre | Queen Elizabeth Hospital |
|  | Blantyre | Mercy James Pediatric Center |
|  | Lilongwe | Kamuzu Central Hospital |
|  | Zomba | Zomba Central Hospital |
| Nicaragua | Managua | Operación Sonrisa Centro de Atención Integral Grace McGregor |
| Venezuela | Caracas | Clínica de Operación Sonrisa Venezuela |
